# Editorial

**DOI:** 10.1093/emph/eou016

**Published:** 2014-04-24

**Authors:** Gillian Bentley

**Affiliations:** Durham University, United Kingdom

*Evolution, Medicine, and Public Health* (*EMPH*) is pioneering a new category of contributions called Clinical Briefs for which we are soliciting further submissions. Briefs are of two types: 1) Clinical—that take an explicitly evolutionary perspective to address a specific condition or pathology and 2) Foundational—that deal with basic topics underpinning an understanding of evolutionary principles that shed light on clinical conditions.

This novel type of publication is restricted to a one-page, 600-word summary, including references and figures, designed to be accessible in style and useful for practitioners. Both kinds of briefs use a standard template with three columns: Clinical Briefs use the first to discuss the targeted pathology, the second to discuss an evolutionary perspective on this pathology and the third to discuss future implications. Foundational Briefs use the first column to give a definition and background to the topic discussed, the second to give relevant examples from human biology and public health and the third to give specific examples from clinical medicine. Briefs can be easily downloaded and read from tablets and mobile phones. As with other contributions to *EMPH*, Briefs are peer-reviewed and searchable online.

Our first Clinical Brief deals with the topics of inflammatory bowel disease (IBD) and fever. In the former, Shona Lee and Rick Maizels (Edinburgh University) point to the strong selection pressures on our immune systems exerted by helminths in our evolutionary past and to the absence of such infections in industrialized societies where rates of IBD are elevated. They discuss how new treatments involving the ingestion of live helminths such as *Trichuris suis* ova—to mimic historic patterns of infectious disease exposure—are reported to reduce IBD symptoms. The second example of a Clinical Brief, written by Elspeth Best (Newcastle University) and Mark Schwartz (NYU School of Medicine), discusses the adaptive function of fever, which occurs across all vertebrate species, and is likely an evolved defence to promote host response to infection. They also summarize the potential negative effects of antipyretics to treat fever and the uncertainty that we do more good than harm by their common use. In the final column, the authors discuss the implications of these findings for future clinical use of antipyretics.

For Foundational Briefs, our first submission is by Peter Ellison (Harvard University), who covers the concept of evolutionary tradeoffs and the implication of these for optimizing health. His example from human biology looks at metabolic energy allocation involving tradeoffs in growth and immune function in children in rural Bolivia. Here, elevated levels of C-reactive protein are linked with lower growth rates. Ellison’s clinical example addresses how hormone replacement therapy in menopausal women may lower the risk of osteoporosis but increase the risk of breast cancer.

We hope you enjoy and benefit from this new and exciting publication format and invite you to submit further contributions.

